# Targeted Genetic Reduction of Mutant Huntingtin Lessens Cardiac Pathology in the BACHD Mouse Model of Huntington's Disease

**DOI:** 10.3389/fcvm.2021.810810

**Published:** 2021-12-24

**Authors:** Saemi Park, Shu Hon Christopher Luk, Raj S. Bains, Daniel S. Whittaker, Emily Chiem, Maria C. Jordan, Kenneth P. Roos, Cristina A. Ghiani, Christopher S. Colwell

**Affiliations:** ^1^Molecular, Cellular and Integrative Physiology Graduate Program, University of California, Los Angeles, Los Angeles, CA, United States; ^2^Department of Psychiatry and Biobehavioral Sciences, David Geffen School of Medicine, University of California, Los Angeles, Los Angeles, CA, United States; ^3^Department of Physiology, David Geffen School of Medicine, University of California, Los Angeles, Los Angeles, CA, United States; ^4^Department of Pathology and Laboratory Medicine, David Geffen School of Medicine, University of California, Los Angeles, Los Angeles, CA, United States

**Keywords:** Huntington's disease, cardiac dysfunction, BACHD mouse model, rescue, hypertrophy, cardiomyopathy, cardiovascular function

## Abstract

Individuals affected by Huntington's disease (HD) present with progressive degeneration that results in a wide range of symptoms, including cardiovascular (CV) dysfunction. The huntingtin gene (*HTT*) and its product are ubiquitously expressed, hence, the cardiomyopathy could also be driven by defects caused by its mutated form (*mHTT*) in the cardiomyocytes themselves. In the present study, we sought to determine the contribution of the *mHTT* expressed in the cardiomyocytes to CV symptoms. We utilized the BACHD mouse model, which exhibits many of the HD core symptoms, including CV dysfunction. This model allows the targeted genetic reduction of *mHTT* expression in the cardiomyocytes while maintaining the expression of the *mHTT* in the rest of the body. The BACHD line was crossed with a line of mice in which the expression of Cre recombinase is driven by the cardiac-specific alpha myosin-heavy chain (*Myh6*) promoter. The offspring of this cross (BMYO mice) exhibited a dramatic reduction in *mHTT* in the heart but not in the striatum. The BMYO mice were evaluated at 6 months old, as at this age, the BACHD line displays a strong CV phenotype. Echocardiogram measurements found improvement in the ejection fraction in the BMYO line compared to the BACHD, while hypertrophy was observed in both mutant lines. Next, we examined the expression of genes known to be upregulated during pathological cardiac hypertrophy. As measured by qPCR, the BMYO hearts exhibited significantly less expression of *collagen1a* as well as *Gata4*, and *brain natriuretic peptide* compared to the BACHD. Fibrosis in the hearts assessed by Masson's trichrome stain and the protein levels of fibronectin were reduced in the BMYO hearts compared to BACHD. Finally, we examined the performance of the mice on CV-sensitive motor tasks. Both the overall activity levels and grip strength were improved in the BMYO mice. Therefore, we conclude that the reduction of *mHtt* expression in the heart benefits CV function in the BACHD model, and suggest that cardiomyopathy should be considered in the treatment strategies for HD.

## Introduction

Patients with Huntington's disease (HD), a progressive degenerative disease, present with cognitive, psychiatric and motor dysfunctions (1, 2). HD is caused by mutations within the first exon of the *huntingtin* (*Htt*) gene located on Chromosome 4, which produce a CAG repeat expansion. Translation of such repeats leads to a polyglutamine (polyQ) repeat with consequent protein misfolding, production of soluble aggregates and inclusion bodies throughout the body (3, 4). The normal function(s) of the HTT protein is still largely unknown and under study; albeit it is thought to be critical for a large range of cellular processes including cytoskeletal organization, protein folding, and metabolic processes (5). Cardiovascular (CV) dysfunction and (mal)events have been reported to play a role in HD progression, and likely to be, among others, one of the leading causes of early death in HD individuals [(6, 7); also see (8, 9)]. The clinical etiology in HD appears to be similar to Parkinson's disease where dysautonomia is a prominent early symptom of the disease (10–12). Furthermore, the mutant huntingtin gene (*mHTT*) is ubiquitously expressed raising the possibility that the observed CV symptoms could be driven by deficits in the cardiomyocytes themselves (13). The relative contribution of the *mHTT* in the heart and brain is difficult to tease apart in a clinical population thus, in the present study, we turned to a model organism to address this issue.

Several animal models of HD, which recapitulate important aspects of the human disease including CV dysfunction (9, 14–16), are available. For example, a classic physiological test of the autonomic nervous system (ANS) function is to measure the baroreceptor reflex in which changes in blood pressure evoke alterations in the heart rate. Both the BACHD (17) and the Q175 (18) lines show clear deficits in such response. Heart rate variability (HRV), a measure of the variation in the beat-to-beat interval, reflects the equilibrium between the sympathetic and parasympathetic systems in the control of heart function(s). Traditionally, it is considered as an indicator of CV health, with low HRV being predictive of CV disease and possibly, mortality (19, 20). Reduced HRV has been observed in the R6/1 (21), BACHD (22), and Q175 (18) models. Therefore, these animal models clearly display the dysautonomia seen in the HD patients.

In addition, there is evidence for direct cardiomyopathy in several of the preclinical models, as shown by the reduced contractility and cardiac output (18, 22–25). Cardiomyocytes appear to have metabolic dysfunction (26) as well as structural abnormalities in the mitochondria (27) that are not likely driven by the ANS. Finally, in *Drosophila*, heart specific expression of *mHtt* resulted in cardiac hypertrophy and decreased contractility (28). Similarly, in mice, when transgenic polyQ expression was driven by a cardiomyocyte specific promotor (α-myosin heavy chain promoter, *Myh6*), heart failure and premature death were observed (29). These two studies provide a proof-of-principle that *mHTT* can drive cardiomyopathy without central nervous system (CNS) involvement, but are subject to the concern that the expression levels were much higher than what is normally observed in HD.

Therefore, as an alternative strategy to explore the impact of heart specific *mHTT* expression on CV pathophysiology, we utilized the BACHD line of mice, which has been engineered to express the human mutation in the *HTT* gene, in a way that it can be excised by Cre recombinase (Cre) in a tissue specific manner (30, 31). Our previous studies showed structural and functional CV changes in the BACHD mice by 6 months of age (17, 22, 32). Therefore, the BACHD mouse was crossed with a line in which the cardiac-specific *Myh6* promoter drives Cre expression only in the cardiomyocytes with the expectation that *mHTT* would be selectively reduced in this cell type. Using this genetically targeted reduction (BACHD X cardiac-specific *Myh6*-Cre: BMYO line), we assessed cardiac function and structure with echocardiograms as well as gene expression of a set of markers associated with pathological hypertrophy in the heart at 6 months of age. Finally, we examined the impact of specific cardiac reduction of *mHTT* on motor behaviors sensitive to CV function. Taken together, our study provides important insights into cardiovascular pathophysiology in HD.

## Methods

### Generation of BMYO Mice

**Figure 1:**
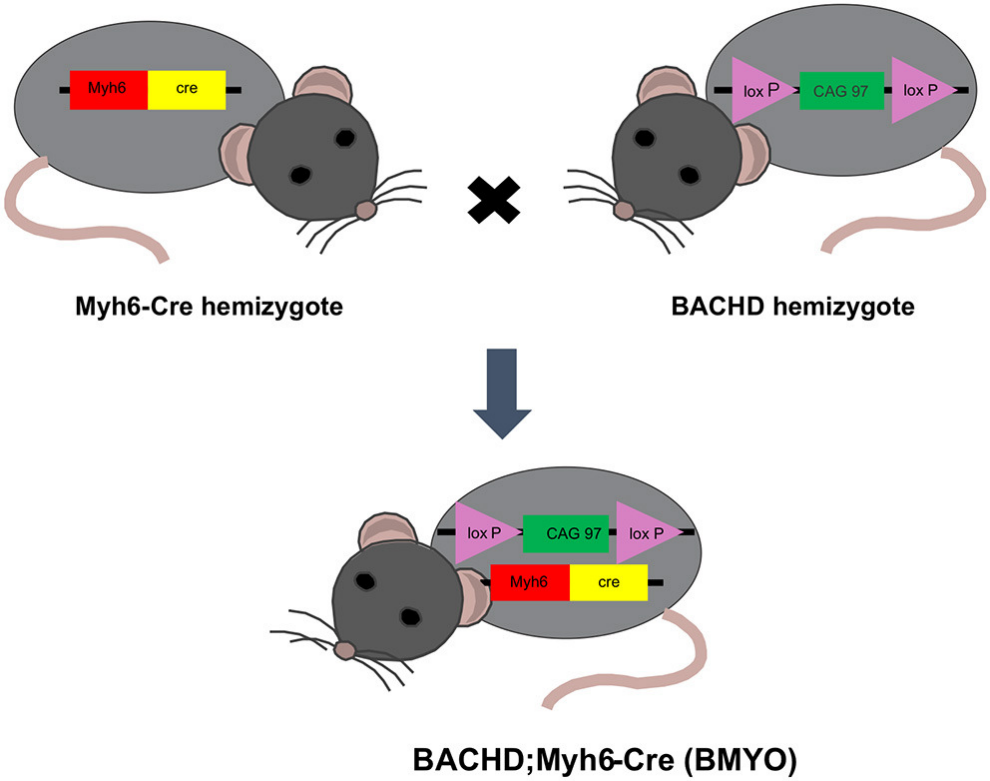
Schematic representation of the breeding strategy used in the present study. Genetic reduction of *mHTT* expression in the heart of BACHD mice was achieved by crossing this transgene (hemizygous) with mice hemizygous for the cardiomyocyte-specific Myh6-Cre transgene. The full-length m*HTT* with two *loxP* sites enables cell specific reduction of m*HTT* using Cre-recombinase (Cre). Approximately 25% of the offspring were double transgenics (BACHD; Myh6-Cre), BMYO, with the *mHTT* floxed out in the cardiomyocytes.

BACHD mice on the C57BL6/J background along with littermate wild-type (WT) controls were acquired from the mouse mutant resource at The Jackson Laboratory, (JAX, Bar Harbor, Maine) in a colony maintained by the CHDI Foundation. α*MHC*^Cre^ (Myh6-Cre) were obtained from the Jackson Laboratory (JAX). BACHD and Myh6-Cre mice on a C57BL/6 background were bred to generate a double transgenic: BACHD; Myh6-Cre (BMYO), with the *mHTT* floxed out in the cardiomyocytes (Figure 1). The following three genotypes were used in this study: WT, BACHD, and BMYO. Genotypes were confirmed by PCR of tail snips.

WT (*n* = 8), BACHD (*n* = 8), and BMYO (*n* = 8) male mice were group-housed and kept in a 12 h:12 h light/dark (LD) cycle with rodent chow provided *ad libitum*. Mice were examined with echocardiograms at 3 and 6 months of age. After the last echocardiogram measurement, the heart and the brain region, striatum, were rapidly dissected on ice and properly stored to be used for subsequent histological and biochemical measurements. The left and right striati were frozen separately to be used for whole tissue protein lysate or total RNA extraction. All procedures followed guidelines of the National Institutes of Health and were approved by the UCLA Animal Research Committee.

### Quantitative Real-Time PCR

WT, BACHD and BMYO mice (3 and 6 months-old; *n* = 3–4 per genotype, per age) were deeply anesthetized with isoflurane, the hearts and striati rapidly dissected, and frozen. Total RNA was extracted using TRIzol^®^ (ThermoFisher; Carlsbad, CA) and following the manufacturer's protocol. The samples were further treated with the Ambion^®^ TURBO DNA-*free*^TM^ (Life Technologies; Waltham, MA), followed by a second extraction with phenol/chloroform. Concentration and purity of the samples were assessed using a ThermoScientific^TM^ NanoDrop^TM^ One Microvolume UV-Vis Spectrophotometer (Canoga Park, CA). Total RNA (600 ng) was reverse transcribed using the iScript^TM^ cDNA Synthesis kit (Bio-Rad Laboratories, Hercules, CA) then analyzed for various transcripts on a CFX Connect^TM^ Real-Time PCR Detection System (Bio-Rad Laboratories). Reactions were setup using the iQ^TM^ SYBR^®^ Green Supermix (Bio-Rad Laboratories) and the QuantiTect^®^ Primer Assay (Qiagen, Valencia, CA). To detect the human *mHTT* expression, primers were designed as previously published (30). The forward primer 5′-ATC TTG AGC CAC AGC TCC AGC CA-3′ recognized both the endogenous mouse HD gene homolog and the exogenous *mHTT*, whilst two reverse primers were designed to be species-specific (human): 5′-GGC CTC CGA GGC TTC ATC AGG-3′; reverse (murine): 5′-TCT GAA AAC ATC TGA GAC TTC ACC AGA-3′. Assays were performed in triplicate following the manufacturer's directions. Negative controls (samples in which reverse transcriptase was omitted) were amplified individually using the same primer sets to ensure the absence of genomic DNA contamination. Amplification specificity was assessed by melting curve. Standard curves were prepared using serial dilutions of control RNA and used for quantification as well as to determine the efficiency of each run. Expression standardization was done using as housekeeping gene *Gapdh* (glyceraldehyde-3-phosphate dehydrogenase; Gene ID 14433; Qiagen). The average values from three technical replicates per sample were normalized to those obtained for *Gapdh* in the same sample and the transcript levels expressed as the mean ± SEM.

### Western Blotting

WT, BACHD and BMYO mice (3 and 6 months-old; *n* = 3–4 per genotype, per age) were deeply anesthetized with isoflurane, the hearts and striati rapidly dissected, and frozen. Whole tissue lysates from the heart and striatum were prepared as previously described with minor modifications (22). Briefly, hearts and striati were homogenized in lysis buffer [50 mM Tris/HCl, 0.25% (w/v) sodium deoxycholate, 150 mM NaCl, 1 mM EDTA, 1nM EGTA, 1% (w/v) Nonidet-P40, 1 mM sodium orthovanadate, 1 mM AEBSF, 10 μg/ml aprotinin, 10 μg/ml leupeptin, 10 μg/ml pepstatin and 4 μM sodium fluoride]. Total protein concentration in cleared extracts was estimated using the Thermo Scientific^TM^ Pierce^TM^ BCA Protein Assay Kit (Thermo Fisher Scientific, Waltham, MA USA). Twenty or thirty-five μg of total proteins were resolved onto a 4–12% Tris-glycine gel (Invitrogen, Carlsbad, CA). Equal protein loading was verified by Ponceau S solution (Sigma, Saint Louis, MO) reversible staining of the membranes as well as later by the relative protein levels of GAPDH (1:3,000, GeneTex, Irvine, CA). Membranes were blocked for 1 hr at room temperature and incubated overnight with the primary antibody diluted in blocking solution [5% (w/v) non-fat milk in PBS containing 0.5% (v/v) Tween-20]. Extracts were analyzed for the relative protein levels of the anti-polyglutamine (polyQ)-expansion disease marker, clone 5TF1-1C2 (mouse monoclonal, 1:1,000, Millipore-Sigma, Temecula, CA), and fibronectin (rabbit polyclonal, 1:500, Millipore-Sigma). Protein bands were detected by chemiluminescence using the ThermoScientific^TM^ Pierce^TM^ SuperSignal West Pico or ECL 2 Western Blotting Substrate with horseradish peroxidase (HRP)-conjugated secondary antibodies (Cell Signaling, Danvers, MA). Relative intensities of the protein bands were quantified by scanning densitometry using the NIH Image Software (Image J, http://rsb.info.nih.gov/ij/). Each background-corrected value was normalized to the according GAPDH levels of the sample and are shown as the mean ± SEM.

### Echocardiogram

WT, BACHD, and BMYO mice (*n* = 8 per genotype) were group housed and kept in a 12:12 LD cycle with rodent chow provided *ad libitum*. Mice were examined with echocardiograms at 3 and 6 months of age. After the last echocardiogram measurement, the heart and brain tissues of the mice were collected and morphologic and histological measurements were taken.

Echocardiograms were obtained using a Siemens Acuson Sequoia C256 instrument equipped with a 15L8 15MHz probe (Siemens Medical Solutions, Mountain View, CA) as previously described (18, 32). Briefly, two-dimensional, M-mode echocardiography and spectral Doppler images enabled measurements of heart dimension and function: [Left Ventricular (LV) Mass], end-diastolic dimension (EDD), end-systolic dimension (ESD), posterior wall thickness (PWT), ventricular septal thickness (VST), LV Ejection Fraction (LV EF). The mice were lightly sedated with 1% isoflurane vaporized in oxygen (Sommi Scientific, Foster City, CA) and heart rate (HR) was monitored using electrocardiogram to maintain physiological levels (between 450 and 600 beats per min).

### Immunofluorescence and Cyto-Histomorphometrical Analyses

Heart samples were harvested from 6 months-old BACHD, perfused with 1x PBS, fixed in 4% paraformaldehyde overnight at 4°C, and cryoprotected in 15% sucrose. After embedding the hearts in Tissue Tek O.C.T. (Sakura Finetek, Torrance, CA), frozen coronal sections (10 μm) were cut on a cryostat (Leica, Wetzlar, GR), sequentially collected on Superfrost Plus glass slides (Thermo Fisher Scientific, Waltham, MA), and stored at −20°C until processed for either immunofluorescence or Masson's Trichrome staining. Mid-ventricular sections slices were identified and paired based on the visual presence of the papillary muscles in the left ventricle.

For immunofluorescence, sections were stained as previously reported with minor modifications (33). Briefly, sections (n = 2 animals/genotype) were blocked in carrier solution [1% (w/v) BSA and 0.3% (w/v) Triton X-100 in 1x PBS] containing 20% (v/v) normal goat serum for 1 h and incubated overnight at 4°C with a rabbit polyclonal antibody against collagen I (abcam, Waltham, MA) diluted in carrier solution containing 5% (v/v) normal goat serum. Sections were washed in carrier solution and then incubated with a goat anti-rabbit secondary antibody conjugated to Cy3 (Jackson ImmunoResearch Laboratories, Bar Harbor, ME). Coverslips applied with a drop of Vectashield mounting medium with DAPI (49,6-diamidino-2-phenylindole; Vector Laboratories, Burlingame, CA). Immunostained sections were visualized on a Zeiss Axio Imager 2 equipped with an AxioCam MRm and the ApoTome imaging system (Carl Zeiss, Germany) using the Zen software (Zeiss).

Masson's Trichrome staining (Sigma-Aldrich, St Louis, MI) was performed as previously reported (18, 22). The presence or absence of fibrotic staining was visually scored by two researchers masked to the experimental conditions in 3 sections/animal. Trichrome stained slices were used for the following cyto- and histo-morphological analyses:

(1) Heart dimensions were measured using 2 sections from each Masson's Trichrome-stained heart. Images were acquired on a Zeiss Stereomicroscope (StemiSV11Apo) equipped with a Zeiss color camera (Axiocam 208 color) using the Zen software (Zeiss). The thickness of the left and right ventricular walls was measured in three non-septal parts to obtain an average thickness. The thickness of the intraventricular septum was determined in three middle parts (non-ventricular). Values from 2 sections/animal (n = 3 per genotype) were averaged.
(2) To measure the cardiomyocytes' cross-sectional area, images from multiple fields of the same 2 sections were acquired at the levels of the papillary of the Lv (10–15 images/animal), from non-septal parts of the Rv wall and the middle intraventricular septum (non-ventricular part; 5–10 images) on a Zeiss upright microscope (Axioskop; 20× objective) equipped with a Zeiss color camera (Axiocam 208 color) and the Zen software (Zeiss). The cross-sectional areas of at least 5–6 cells/image/animal were averaged and analyzed for statistical difference (*n* = 3 per genotype).
(3) The number of cells was determined in the same images acquired for the analysis of the cross-sectional areas, using the cell counting plugin/feature of ImageJ by an observer masked to the genotype. Cells were counted in 2–3 ROI (regions of interest) of equal size (153 μm × 153 μm) in each image. The number of cells/image was summed, and then divided by the total area of the 2 or 3 ROIs. The values from 10–15 images (Lv) or 5–10 images (Rv and Septum)/animal were averaged and analyzed for statistical difference. The number of cells/μm^2^ is reported (*n* = 3 per genotype).

All the morphological assessments were performed using Image J by an observer masked to the genotype.

### Behavioral Studies

Locomotor activity was recorded for 2 weeks from single-caged mice with infrared motion sensors using our established protocols (34). Locomotor activity was recorded in 3-min bins, and 10 days of data were averaged for analysis. The amount of cage activity over a 24-h period was averaged over 10 days and reported here as the hourly arbitrary units (au/day). The activity data was also analyzed for possible differences in diurnal rhythmicity. The period and rhythmic strength was determined as previously described (34, 35). The periodogram analysis used a χ^2^ test with a threshold of 0.001 significance, from which the amplitude of the periodicities was determined at the circadian harmonic to obtain the rhythm power. The number of activity bouts and the average length of bouts were determined using Clocklab (Actimetrics, Wilmette, IL), where a bout consists of a duration in which activity never falls below the criterion (3 counts/min) for longer than the maximum gap (21 min).

The grip strength test was used to measure neuromuscular function as maximal muscle strength of forelimbs. The grip strength ergometer (Santa Cruz Biotechnology, Santa Cruz, CA) was set up on a flat surface with a mouse grid firmly secured in place. Peak mode was utilized to enable measurement of the maximal strength exerted. Mice were tested in their active phase under dim red light (3 lux), and prior to testing, mice were acclimated to the testing room. Mice underwent five trials with an inter-trial interval of at least 2 mins. For each trial, a mouse was lowered slowly over the grid, and only its forepaws were allowed to grip the grid. While the mouse was steadily pulled back, the experimenter ensured the mouse remained horizontal until the mouse was no longer able to grip the grid. The maximal grip strength value of the mouse was recorded each trial. The apparatus was cleaned with 70% ethanol and allowed to dry before testing each mouse cohort. The maximum value obtained per animal is reported as maximal strength divided by bodyweight.

### Statistics

We were interested in determining if the reduction of *mHTT* would improve the symptoms reported in the BACHD mouse model; therefore, the BMYO mice were compared to age-matched BACHD and WT mice in all the experiments. The sample size per group was determined by both our empirical experience with the variability in the prior measures in the BACHD mice. In addition, for the echocardiograms and behavioral analysis, we carried out a power analysis that assumed a power of 0.8 and an alpha of 0.05. One-way ANOVA along with Holm-Sidak or Tukey's *post-hoc* test was used to evaluate the statistical significance of the findings. Data were examined for normality (Shapiro-Wilk test) and equal variance (Brown-Forsythe test). If normality or equal variance tests failed, then the Kruskal-Wallis one-way analyses of variance (ANOVA) on ranks followed by Dunn's Multiple comparison test was used instead. Statistical analysis was performed using SigmaPlot (SYSTAT Software, San Jose, CA) or Prism 9 (Version 9.2.0; GraphPad Software, La Jolla, CA). Between-group differences were determined significant if *P* < 0.05. All values are reported as group mean ± standard error of the mean (SEM).

## Results

### Genetic Reduction of *mHTT* in the Cardiomyocytes

**Figure 2:**
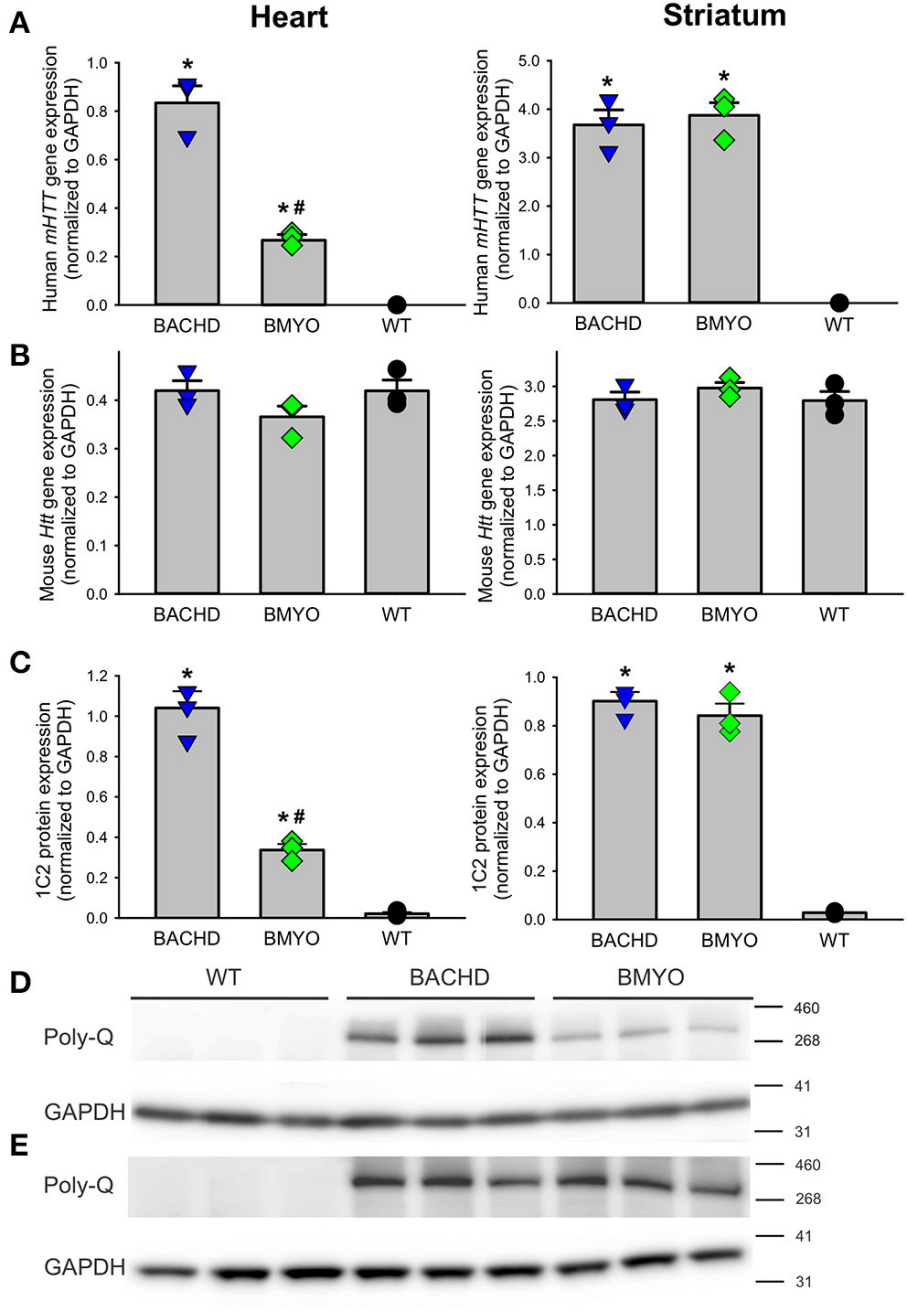
Genetic targeting of human mHTT elicits a selective reduction of its transcript and protein expression levels in the heart, but not the striatum of 3 months old BMYO mice. **(A)** Quantification of the human *mHTT* transcript levels in the heart and striatum shows a significant and selective decrease in the heart of the BMYO line [*F*_(2, 8)_ = 125.5, *P* < 0.001]; while, striatal levels were comparable in the two mutant lines [*F*_(2, 8)_ = 87.706, *P* < 0.001]. **(B)** The levels of the endogenous mouse *Htt* were unchanged among genotypes in both the heart [*F*_(2, 8)_ = 2.045, *P* = 0.210] and striatum [*F*_(2, 8)_ = 0.852, *P* = 0.473]. **(C)** PolyQ protein expression levels were detected in the BACHD heart and were greatly reduced in the BMYO [*F*_(2, 8)_ = 162.549, *P* < 0.001], but not in the striatum of both mutants. These data demonstrate that the genetic cross was successful, and the *mHTT* levels were selectively reduced in the BMYO heart, while maintaining “normal” expression in the striatum. Representative immunoblots for the heart **(D)** and **(E)** striatum. Values derived from the densitometric analysis were corrected for the background and normalized to GAPDH. The vertical bar plots represent the group means and SEM. The symbols show the values for each individual animal (n = 3) per genotype. Asterisks represent significant differences (*P* < 0.05) compared to WT while crosshatch indicates significant differences (*P* < 0.05) between BMYO and BACHD. One way ANOVA followed by Tukey's multiple comparison test was used to evaluate the possible significance of the findings.

To determine whether genetically reducing *mHTT* expression from the cardiomyocytes would rescue the cardiovascular phenotypes observed in the BACHD mice, we crossed BACHD mice with Myh6-Cre mice and generated the BACHD;Myh6-Cre (BMYO) (Figure 1). A significant reduction in the *mHTT* transcript **(**67% as compared to the BACHD; Tukey: *q* = 18.584, *P* < 0.001**)** expression levels was observed in the heart of 3 months old BMYO animals compared to the BACHD, but not in the striatum (Figure 2A). No differences were found in the expression of the endogenous *Htt* in the heart (Figure 2B) between the three mouse groups. Furthermore, we assessed the protein levels of mHTT in whole heart and striatum tissue lysate by using an antibody that recognizes the polyglutamine-expansion (polyQ) present in the pathological form of this protein. Significant differences in the polyQ expression levels were observed among the genotypes, with the BMYO displaying a significant reduction (65% as compared to the BACHD; Tukey: *q* = 20.801, *P* < 0.001; Figures 2C,D). In the BMYO striatum, the polyQ expression was unaffected and at a similar expression level as in the BACHD (Figure 2C). In summary, the BMYO hearts displayed a significant reduction in mHtt at both the transcript and protein level as compared to the BACHD hearts, while its expression was not affected in the striatum.

### Genetic Reduction of the *mHTT* Expression Improves Some Cardiovascular Functions in BMYO Mice

**Figure 3:**
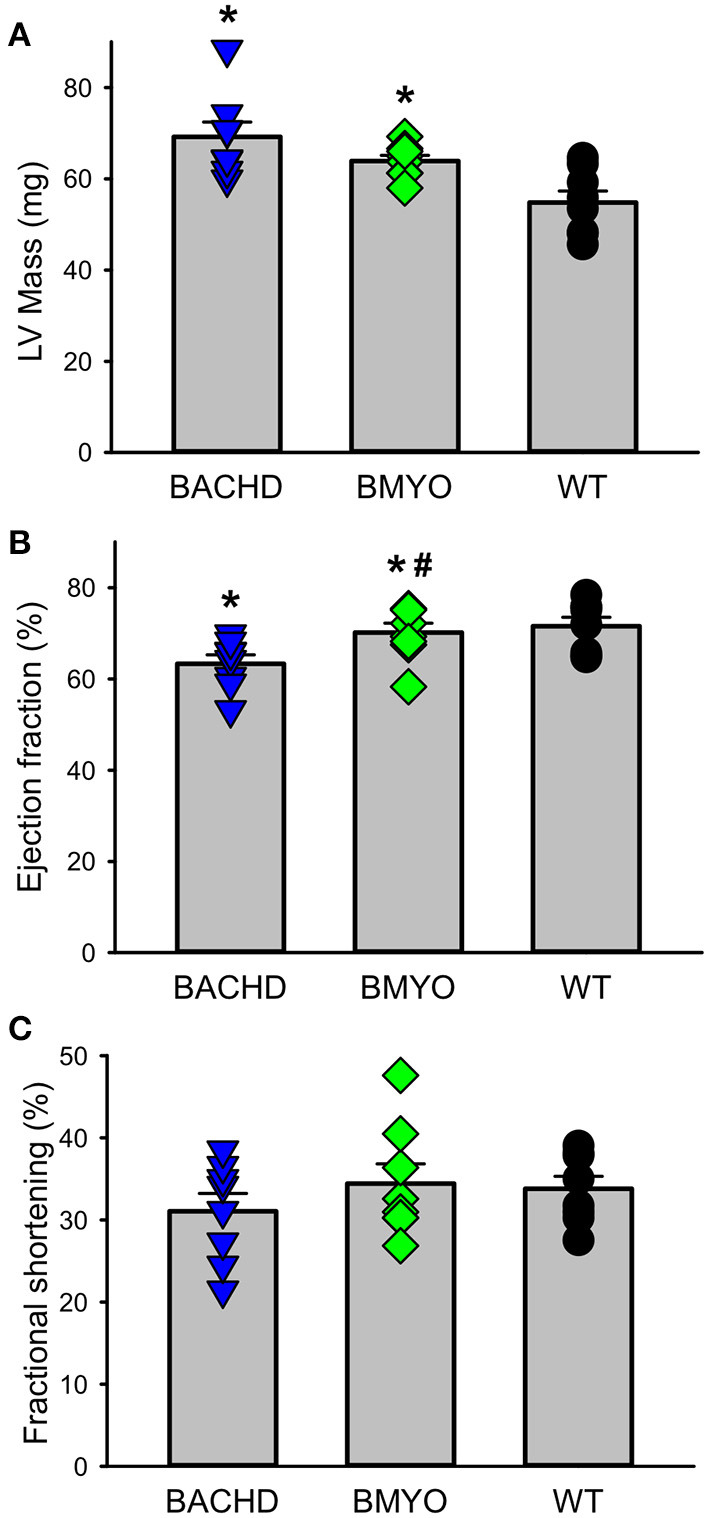
Echocardiographic measurements of BACHD, BMYO, and WT animals at 6 months of age. Two-dimensional, M-mode echocardiography and spectral Doppler images enabled measurement of heart dimension and function including Left ventricle mass (LV mass), fractional shortening (FS%), Lv Ejection Fraction (Lv EF), and heart rate. Most parameters did not vary between the genotypes (Table 1). **(A)** Lv mass was increased in both the BACHD and BMYO mice compared to WT controls, while **(B)** the ejection fraction was reduced in BACHD compared to BMYO and WT hearts. **(C)** The fractional shortening followed a similar pattern although the differences were not significant by our criterion. The selective reduction of mHtt from the heart did not reduce the hypertrophy in the BMYO heart but seemed to improve the ejection fraction. The vertical bar plots show group means and SEM. The symbols show the values from individual animals (*n* = 7–8 per genotype). Asterisks represent significant differences (*P* < 0.05) compared to WT controls while crosshatch indicates significant differences (*P* < 0.05) between BMYO and BACHD. One way ANOVA followed by Tukey's multiple comparison test was used to evaluate the possible significance of the findings (see Table 1).

The effects elicited by the reduction of *mHTT* on the cardiac structure and functions were assessed by echocardiogram at 3 and 6-month age. Consistent with our earlier data (22) showing little loss of cardiac function in the BACHD mice at 3 months of age, few significant differences could be observed among the three genotypes (Table 1). At this age, the Lv of both mutants exhibited an increased mass (not significant), but only the BACHD exhibited a reduced ejection fraction (Tukey: *q* = 3.941, *P* = 0.029) compared to WT. The fractional shortening (FS, %) was just under with the BACHD showing reduced values compared to the BMYO and WT groups. At 6 months of age (Figure 3; Table 1), the BACHD (Tukey: *q* = 5.809, *P* = 0.002) and the BMYO (Tukey: *q* = 3.664, *P* = 0.043) exhibited significantly enlarged left ventricles compared to WT. Even though such feature clearly was not altered by the removal of the *mHTT*, still it is notable that the mass in the BMYO mass did not further increase compared to the measurement at 3 months old. Most of the other parameters did not vary among the genotypes. On the other hand, the ejection fraction (EF) was significantly reduced compared to WT in the BACHD (Tukey: *q* = 4.889, *P* = 0.006), while did not significantly change in the BMYO. The BMYO mice display a heart functionality closer to the WT. Overall, at the two ages points examined, most of the echocardiogram parameters did not vary significantly. The measure that appeared to be the most sensitive to the levels of *mHTT* in the cardiomyocytes was the EF, which refers to the percentage of blood that is pumped out of a filled ventricle with each heartbeat.

**Table 1:** Echocardiographic parameters in BACHD, BMYO and WT animals at 3 and 6 months of age.

	**BACHD**	**BMYO**	**WT**	**Stats**
	**Mean ± SEM**			
**Age (months)**	**3**	**3**	**3**	
Lv mass (mg)	60 ± 5	63 ± 4	52 ± 4	*F*_(2, 23)_ = 1.784; *P* = 0.193
FS (%)	30 ± 2	35 ± 1	37 ± 2	*F*_(2, 23)_ = 3.186; *P* = 0.062
E/A	1.9 ± 0.1	2.0 ± 0.1	2.0 ± 0.1	*F*_(2, 23)_ = 0.027; *P* = 0.973
Lv EF	63 ± 2	68 ± 1	71 ± 3	***F*** **_(2, 23)_ = 3.952; ***P*** = 0.035**
**Age (months)**	**6**	**6**	**6**	
Lv mass (mg)	69 ± 3	64 ± 2	55 ± 2	***F*****_(2, 23)_ = 5.210;** ***P*** **= 0.015**
FS (%)	28 ± 2	32 ± 1	34 ± 1	*F*_(2, 23)_ = 3.148; *P* = 0.064
E/A	1.9 ± 0.1	2.0 ± 0.1	2.0 ± 0.1	*F*_(2, 23)_ = 0.027; *P* = 0.973
Lv EF	63 ± 2	69 ± 2	71 ± 2	***F*****_(2, 23)_ = 6.689;** ***P*** **= 0.006**

*Two-dimensional, M-mode echocardiography and spectral Doppler images enabled measurement of heart dimension and function including Left ventricle mass (Lv mass), ratio of the early (E) to late (A) ventricular filling velocities (E/A ratio), fractional shortening (FS%), Lv Ejection Fraction (Lv EF). For these data and those shown in the other tables, one-way ANOVA was used to evaluate the possible significance of the findings. If normality or equal variance tests failed, then a Kruskal-Wallis one way ANOVA on ranks was used instead. Bold values indicate statistically significant genotypic differences. N = 7–8 per genotype, all males*.

### The Reduction of mHTT Impacted Gene Expression for Cardiovascular Disease Markers Within the BMYO Hearts

**Figure 4:**
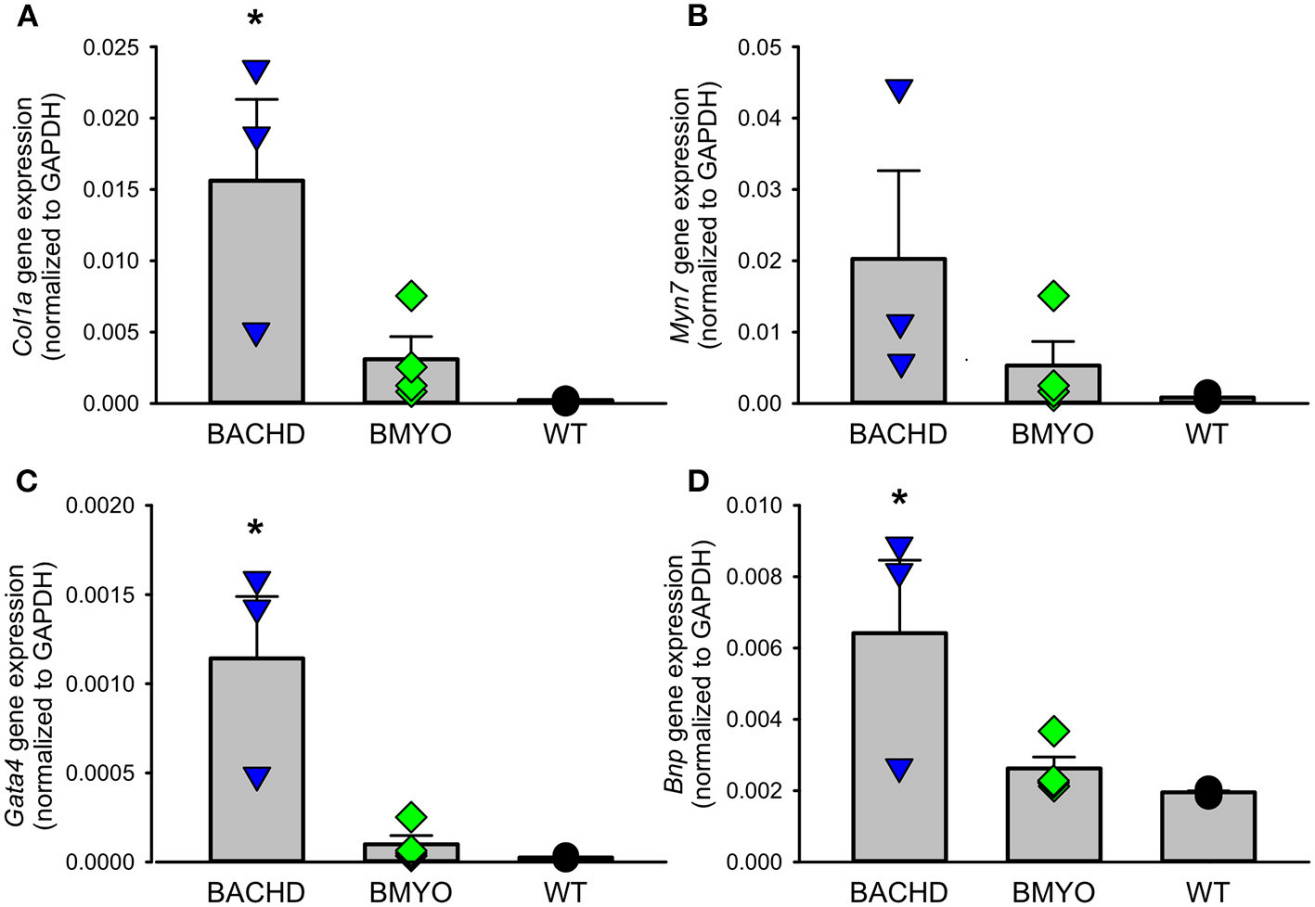
Gene expression levels of molecular markers for cardiovascular injury and repair were significantly lowered by the specific removal of *mHtt* from the heart. **(A)** The expression levels of collagen 1a (*Col1a*) were significantly lower in the BMYO animals compared to the BACHD, while **(B)** the expression of ventricular/slow myosin heavy chain isoform (*Myh7*) did not significantly change. **(C)** GATA binding protein 4 (*GATA4*) and **(D)** brain natriuretic peptide (*BNP*) were both significantly diminished in the BMYO heart at 6 months of age as compared to BACHD (see also Table 2 for values derived from the statistical analyses). Together these data suggest that changes in molecular markers of CV injury were reversed by selective removal of the *mHTT* in the BMYO line. The vertical bar plots show group means and SEM. The symbols show the values from individual animals (n = 3–4 per genotype). Asterisks represent significant differences (*P* < 0.05) compared to WT controls. One way ANOVA followed by Tukey's multiple comparison test was used to evaluate the possible significance of the findings. If normality or equal variance tests failed, then Kruskal-Wallis one-way ANOVA on ranks followed by Dunn's multiple comparison test was used instead (see also Table 2).

**Figure 5:**
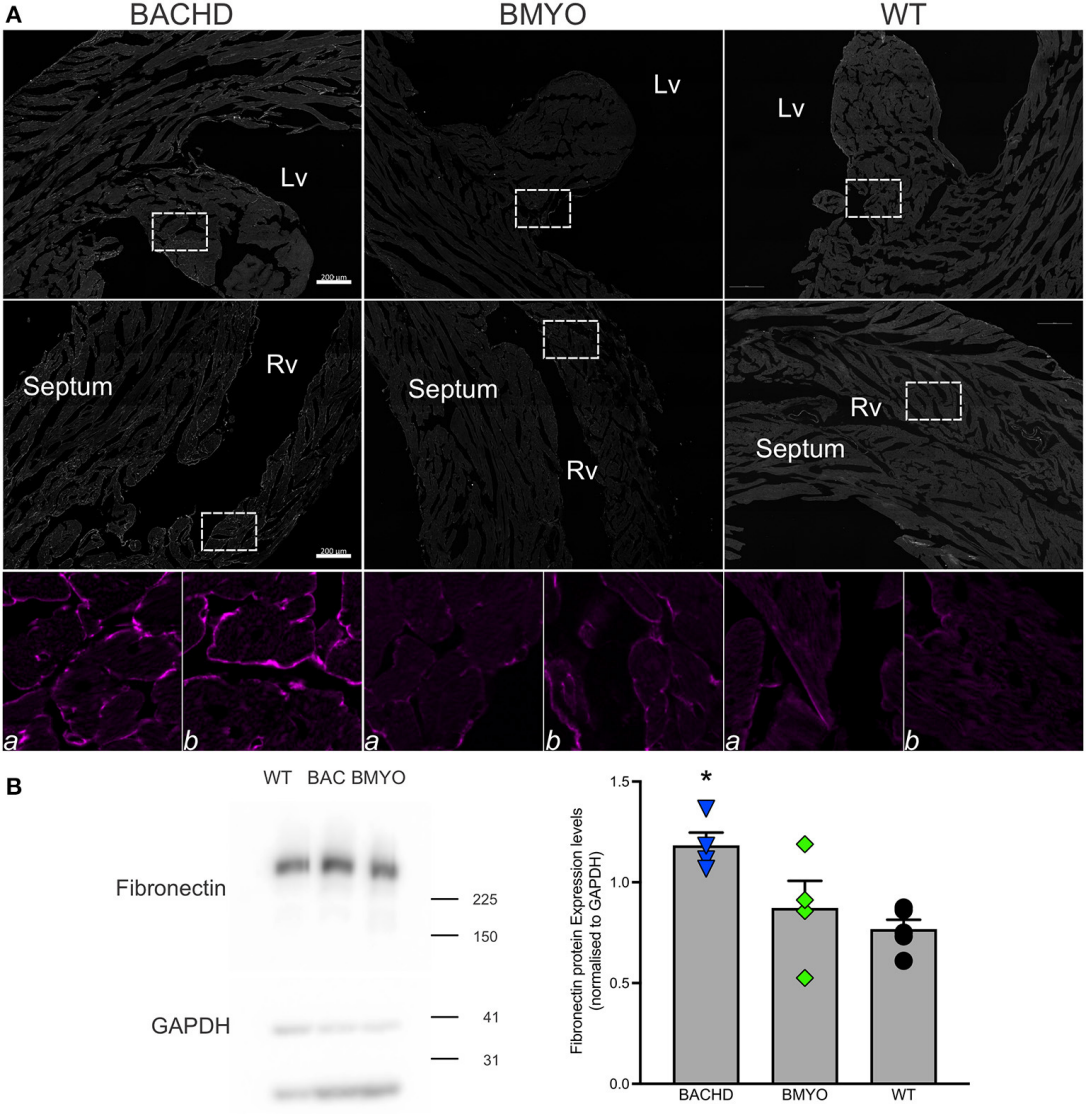
The expression of fibrotic markers was diminished by the selective removal of human *mHTT* from the cardiomyocytes. **(A)** Representative images of immunostained mid-ventricular coronal heart sections showing stronger and diffuse expression of Collagen I in 6 months-old BACHD, but not in age-matched BMYO and WT mice (*n* = 2 per genotype). The white rectangles delineate the areas where the enlarged images were obtained: (a) left ventricular wall-papillary muscle; (b) right ventricular wall. Intense collagen I immunoreactivity can be observed around the cardiomyocytes in the BACHD heart (left panels), but at a lower level in the BMYO and WT (middle and right panels, respectively). Lv, left ventricle; Rv, right ventricle. Scale bars = 200 μm. **(B)** Representative western blotting of whole-tissue lysates of hearts from 6 months-old BACHD, BMYO and WT mice. The total protein levels of fibronectin displayed a significant increase in the BACHD mice as compared to WT. Each value derived from the densitometric analysis was corrected for background, and then divided by the sum of the background-corrected signals obtained in the same immunoblot. These values were then normalized to the respective relative GAPDH levels, which were corrected in the same manner. The vertical bar plots represent the group means and SEM. The symbols show the values for each individual animal (n = 4–5) per genotype. Kruskal-Wallis one-way ANOVA on ranks (*H* = 6.003; *P* = 0.042) followed by Dunn's multiple comparison test (*P* = 0.045) was used to evaluate the possible significance of the findings. Asterisks represent significant differences (*P* < 0.05) compared to WT mice.

Along with abnormal echocardiogram parameters, a pathophysiological feature of the middle-aged (>12 months) BACHD hearts is to display extensive fibrotic tissue (22); however, it is unknown how early such process presents. Thus, the levels of transcripts known to be specific markers for cardiovascular disease were analyzed in the mutant hearts at 6 months of age (Figure 4; Table 2). The expression levels of the marker for cardiac fibrosis collagen 1a (*Col1a*) were found significantly augmented in the BACHD hearts as compared to the WT (Tukey: *q* = 4.710, *P* = 0.03), while the BMYO showed no significant difference from the other groups (Figure 4A; Table 2). The ventricular/slow myosin heavy chain isoform (*Myh7*), a gene associated with increased fibrosis and hypertrophic cardiomyopathy (36), showed a trend to increase in the BACHD (Figure 4B), with no significant changes in the BMYO in comparison to the WT. It is well established that the expression of genes involved in cardiomyocyte development is up-regulated in response to injury or myocardial remodeling in response to physiological and pathological events (e.g. 32–34). We found a significant increase in the expression of the GATA binding protein 4 (*Gata4*) in the hearts of the BACHD (Dunn's Method: Q = 2.697, *P* = 0.021 vs. WT) which was absent in the BMYO line (Figure 4C; Table 2). In addition, the BACHD mice exhibited elevated transcript levels of brain natriuretic peptide (*Bnp*, Figure 4D; Table 2; Dunn's Method: Q = 2.562, *P* = 0.031 vs. WT), a hormone secreted by cardiomyocytes in the ventricles in response to stretching. Again, the BMYO line showed a much lower expression. In agreement, increased Collagen I immunoreactivity could be observed in mid-ventricular coronal sections of 6 months of age BACHD, in particular along the septal and Rv walls, with the BMYO hearts showing a much lower staining (Figure 5A). Finally, the protein levels of fibronectin were significantly elevated in the BACHD heart in comparison to the WT (*H* = 6.003; *P* = 0.042, Kruskal-Wallis one-way ANOVA on ranks followed by Dunn's multiple comparison test, *P* = 0.045), and consistently reduced in the BMYO heart lysates (Figure 5B). Altogether these findings suggest that the BACHD hearts are under stress and such changes appear to be prevented by the selective deletion of the human *mHTT* from the cardiomyocytes of the BMYO mice.

**Table 2:** expression levels of selected genes in the hearts of BACHD, BMYO and WT animals at 6 months of age.

**Gene**	**Gene ID**	**BACHD**	**BMYO**	**WT**	**Stats**
		**mean ± SEM (normalized to Gapdh)**			
*Col1a*	12842	0.0156 ± 0.005	0.0031 ± 0.003	0.0002 ± 0.0001	***F*****_(2, 9)_ = 6.410;** ***P*** **= 0.026**
*Myh7*	140781	0.020 ± 0.012	0.005 ± 0.003	0.001 ± 0.0003	*F*_(2, 9)_ = 2.095; *P* = 0.194
*Gata4*	14463	0.0011 ± 0.0003	0.00001 ± 0.00004	0.00002 ± 0.000006	***H*** **= 7.318;** ***P*** **= 0.004**
*Bnp*	18158	0.006 ± 0.002	0.003 ± 0.0003	0.002 ± 0.0003	***H*** **= 6.745;** ***P*** **= 0.010**

*Primers targeting the collagen 1a (Col1a); the ventricular/slow myosin heavy chain isoform (Myh7); the GATA binding protein 4 (Gata4), and the brain natriuretic peptide (Bnp) were used. For each gene, the average values from three technical replicates per sample were normalized to those obtained for Gapdh in the same sample. Bold values indicate statistically significant genotypic differences. N = 3–4 per genotype, all males*.

### Reduction of *mHTT* Lessens Fibrosis

**Figure 6:**
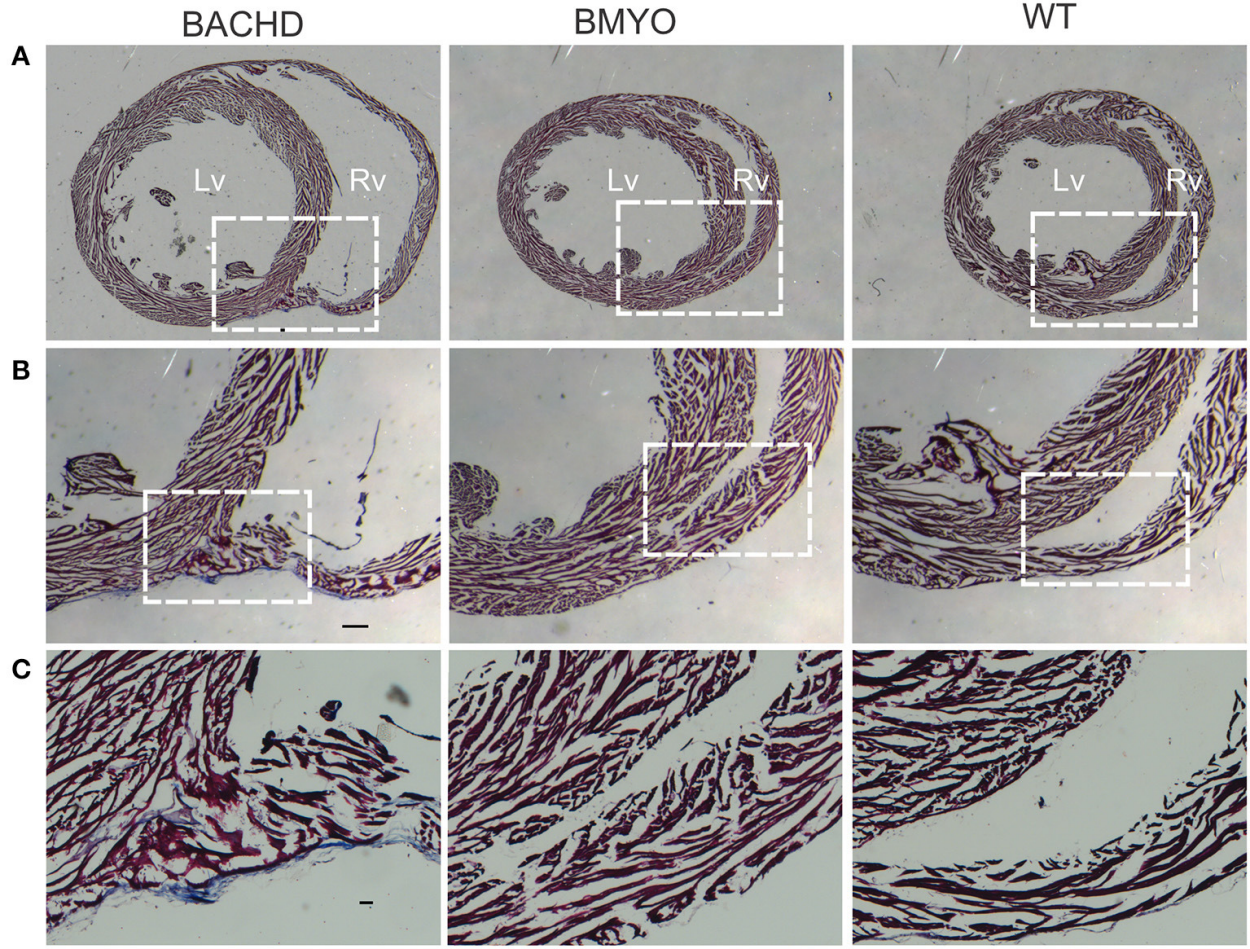
Selective removal of the human *mHtt* lessened the fibrosis in the hearts of 6 months old BMYO mice. Representative photomicrographs of mid-ventricular heart sections stained with Masson's trichrome at different magnifications. For all three genotypes, a representative stained section is shown at three progressively higher magnifications. **(A)** Lower magnification images showing the enlarged BACHD heart, such phenotype was less evident in the BMYO mice (scale bar = 200 μm). The white rectangles delineate the areas where the higher magnification images were acquired. **(B,C)** Higher magnification images showing pockets of fibrotic tissue of variable extension in the septal part of the right ventricular wall of BACHD hearts. Some fibrotic areas could be also seen along the outer surface of the ventricular walls near the interventricular septum. Conversely, such areas were qualitatively judged to be almost inexistent in the BMYO (Table 3). At this age, WT hearts did not show strong evidence of fibrosis. Scale bars = 100 and 50 μm in the middle and lower panels, respectively. *n* = 3 per genotype. Lv, left ventricle; Rv, right ventricle.

As mentioned above, we have previously reported that 12 months old BACHD mice (22) present with an enlarged and fibrotic heart. Hence, in the present study, Masson's Trichrome staining was used to investigate the presence of fibrotic tissue, as well as cyto-histomorphological abnormalities in 6 months old BACHD and BMYO hearts. Whilst fibrotic staining was almost undetectable in the WT hearts (1/3 mice), all the BACHD hearts (3/3 mice) exhibited some pockets of fibrosis of variable extension located mostly in correspondence of the septal parts of the Rv wall (Figures 6B,C). The BMYO hearts (3/3 mice) appeared to have fewer and quite small fibrotic areas compared to what observed in the BACHD (Table 3).

**Table 3:** Masson's Trichrome staining was used to investigate the presence of fibrotic tissue in heart sections from WT, BACHD and BMYO mice at 6 months of age.

	**Lv wall and Papillary muscles**	**Septum**	**Rv wall**
BACHD	+	++	++
BACHD	+	++	++
BACHD	+	++	++
BMYO	+	+	+
BMYO	−−	+	+
BMYO	+	+	++
WT	−−	−−	−−
WT	−−	−−	−−
WT	+	+	+

*Sections (3/animal) at the mid-ventricular level were chosen based on the visual presence of the papillary muscles in the left ventricle (Lv). Two observers independently evaluated the presence and degree of fibrotic staining in the left ventricular wall and papillary muscles, right ventricular wall (Rv) as well as in the interventricular septum. The degree of fibrotic staining was classified as moderate (++), weak (+), or none (−−) in 3 sections/animal; n = 3 animals of each genotype*.

To further assess possible morphological variations, including changes in cell numbers or cell morphology, which could account for the enlarged heart observed in BACHD mice, we performed a series of measurements. Albeit not significant, the BACHD hearts appeared enlarged while the BMYO hearts were sized similarly to WT (Table 4). Whilst no differences were observed in the Lv of the three genotypes, strikingly, the Rv of the BACHD, but not of the BMYO, displayed a thinner wall and an enlarged lumen (Figure 6A; Table 4). The cross-sectional area or any other cytological measurement were not different among the three groups; however, an increased number of cardiomyocytes was observed in the Rv of the BACHD (Table 4). Hence, the BACHD mice exhibited cyto-histopathological changes also early in disease progressions, with a larger and thinner heart compared to the BMYO as well as the WT mice. Thus, genetically removing the *mHTT* from the cardiomyocytes may improve the defects observed in the BACHD hearts.

**Table 4:** Gross morphological measurements were performed in heart coronal sections from WT, BACHD and BMYO mice at 6 months of age.

	**BACHD**	**BMYO**	**WT**	**Stats**
**Whole heart**				
Cross sectional area (mm^2^)	36.9 ± 4.31	33.0 ± 1.40	32.5 ± 2.37	*H* = 0.932; *P* = 0.667
Circumference (mm)	22.3 ± 1.92	20.8 ± 0.75	20.7 ± 0.77	*H* = 0.800; *P* = 0.721
**Left ventricle**				
Wall thickness (mm)	0.88± 0.01	0.80 ± 0.06	0.87 ± 0.01	*H* = 0.621; *P* = 0.829
Lumen cross-sectional area (mm^2^)	11.5 ± 1.65	12.6 ± 1.20	11.1 ± 0.51	*H* = 1.867; *P* = 0.438
Lumen perimeter (mm)	17.4 ± 1.48	17.6 ± 0.72	15.3 ± 1.34	*H* = 1.689; *P* = 0.511
Lumen diameter (mm)	4.52 ± 0.23	4.73 ± 0.25	4.42 ± 0.10	*H* = 1.156; *P* = 0.629
Average Cell number/μm^2^	0.0029 ± 0.00027	0.0033 ± 0.00009	0.0032 ± 0.00018	*H* = 1.867; *P* = 0.439
Total Cell number/μm^2^	0.020 ± 0.004	0.026 ± 0.006	0.024 ± 0.001	*H* = 0.622; *P* = 0.829
Cell cross-sectional area (μm^2^)	99.9 ± 3.55	95.0 ± 2.56	123.4 ± 12.7	*H* = 5.067; *P* = 0.086
Cell diameter (μm)	4.83 ± 0.09	4.55 ± 0.03	5.30 ± 0.29	***H*** **= 5.956;** ***P*** **= 0.025**
Cell perimeter (μm)	13.7 ± 0.38	13.3 ± 0.35	16.2 ± 0.92	***H*** **= 5.956;** ***P*** **= 0.025**
**Septum**				
Wall thickness (mm)	0.81 ± 0.07	0.71 ± 0.01	0.80 ± 0.06	*H* = 1.689; *P* = 0.511
Average Cell number/μm^2^	0.0033 ± 0.00013	0.0034 ± 0.00006	0.0035 ± 0.00018	*H* = 0.356; *P* = 0.879
Total cell number/μm^2^	0.020 ± 0.004	0.026 ± 0.006	0.024 ± 0.001	*H* = 0.622; *P* = 0.829
Cell cross-sectional area (μm^2^)	108.8 ± 3.06	108.2 ± 4.18	110.5 ± 3.44	*H* = 0.089; *P* = 0.993
Cell diameter (μm)	16.2 ± 0.35	16.2 ± 0.75	16.6 ± 0.18	*H* = 1.689; *P* = 0.511
Cell perimeter (μm)	44.6 ± 0.60	44.6 ± 0.61	45.2 ± 0.19	*H* = 0.267; *P* = 0.929
**Right ventricle**				
Wall thickness (mm)	0.31 ± 0.04	0.40 ± 0.04	0.42 ± 0.08	*H* = 2.489; *P* = 0.338
Lumen cross-sectional area (mm^2^)	6.61 ± 2.85	3.12 ± 1.75	3.09 ± 1.97	*H* = 1.689; *P* = 0.511
Lumen perimeter (mm)	17.9 ± 2.28	14.8 ± 0.81	13.6 ± 1.84	*H* = 2.222; *P* = 0.381
Average cell number/μm^2^	0.0033 ± 0.00018	0.0029 ± 0.00004	0.0028 ± 0.00003	***H*** **= 5.535;** ***P*** **= 0.050**
Total Cell number/μm^2^	0.025 ± 0.008	0.022 ± 0.003	0.020 ± 0.003	*H* = 0.157; *P* = 0.950
Cell cross-sectional area (μm^2^)	108.9 ± 1.20	108.1 ± 8.26	98.7 ± 14.7	*H* = 0.267; *P* = 0.929
Cell diameter (μm)	16.7 ± 0.45	15.9 ± 1.05	15.7 ± 0.71	*H* = 1.156; *P* = 0.629
Cell perimeter (μm)	44.7 ± 1.08	43.2 ± 2.12	42.9 ± 2.28	*H* = 0.267; *P* = 0.929

*Sections (2/animal) at the mid-ventricular level were chosen based on the visual presence of papillary muscles in the left ventricle (Lv). Since the left and right ventricles (Rv) were not perfectly round, we refer to the length of the lumen as perimeter. Cells were counted in 2-3 ROI of equal size (153.21 μm × 153.21 μm)/image and summed. The sums from 10-15 images (Lv) or 5-10 images (Rv and Septum)/animal were averaged and then divided by the ROI total area. Values are presented as the Mean ± SEM, statistical differences were determined using the Kruskal-Wallis one way ANOVA on ranks followed by Dunn's multiple comparisons test. Bold values indicate statistically significant genotypic differences. n=3 animals per genotype, all males*.

### Reduction of *mHTT* in the Heart Improved Some Aspects of Motor Behavior

**Figure 7:**
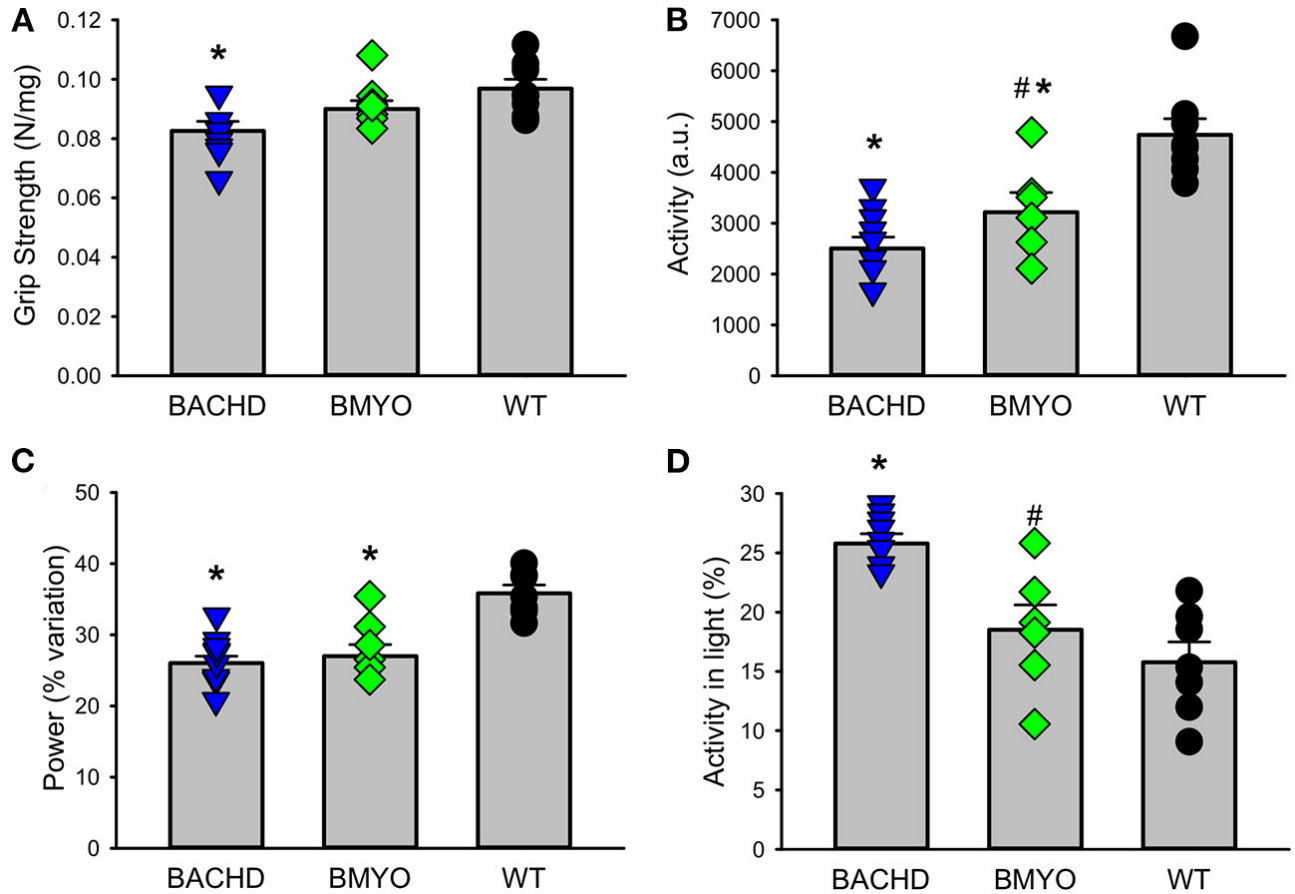
Behavioral biomarkers of cardiovascular function were improved in the BMYO line. Behavior measures including grip strength, and diurnal rhythms of activity of the three lines were measured between 6 and 7 months of age. **(A)** Grip strength was reduced in the BACHD compared to BMYO and WT lines. **(B)** The average total activity over 24 h as reduced in both the BACHD and BMYO compared to WT. **(C)** Power, one measure of circadian rhythmicity, was reduced in both the BACHD and BMYO compared to WT controls. **(D)** The % of activity measured in the light was reduced in the BMYO. The vertical bar plots show group means and SEM. The symbols show the values from individual animals (n = 8 per genotype) in each group. Asterisks represent significant differences (*P* < 0.05) compared to WT mice while crosshatch indicates significant differences (*P* < 0.05) between BMYO and BACHD mice. One way ANOVA followed by Holm-Sidak or Tukey's multiple comparison test was used to evaluate the possible significance of the findings. If normality or equal variance tests failed, then Kruskal-Wallis one way ANOVA on ranks followed by Dunn's multiple comparison test was used instead (see Table 5).

There is compelling evidence that a reduction in physical activity is a sensitive biomarker for chronic CV disease (37, 38). Therefore, in our final experiment, we sought to determine whether the genetic reduction of *mHTT* expression in the cardiomyocytes had beneficial impact on motor function in the BACHD mice using two measures of motor function: grip strength and overall physical activity in the 24-hr cycle (Figure 7; Table 5). Grip strength was improved in the 6 months old BMYO mice compared to BACHD alone (Figure 7A; Holm-Sidak: *t* = 3.262, *P* =0.011 BACHD vs. WT). In addition, both BACHD and BMYO exhibited a significant reduction in activity over 24 h compared to WT (Tukey: BACHD, *q* = 8.850, *P* < 0.001; BMYO, *q* = 6.613, *P* < 0.001), although the BMYO mice still exhibited more activity than the BACHD (Figure 7B). The strength of the daily rhythms as measured by power of the periodograms was similar in both mutant lines, so the rhythmicity of the activity data was not impacted by the reduction in *mHTT* in the heart (Figure 7C). BACHD mice have been shown to be more active in the day, during their normal rest time than WT (32, 39). Unexpectedly, such inappropriate activity in the day was improved in the BMYO line compared to BACHD (Figure 7D; Holm-Sidak: *t* = 3.348, *P* = 0.007). Overall, the performance of the mice on two motor tasks (grip strength, total activity) was improved by reducing *mHTT* in the heart.

**Table 5:** Behavioral measurements of motor function from BACHD, BMYO and WT animals at 6 months of age.

	**BACHD**	**BMYO**	**WT**	**Stats**
	**Mean** **±** **SEM**			
Body weight	25.1 ± 0.7	25.4 ± 2.4	26.7 ± 1.7	*F*_(2, 23)_ = 0.245; *P* = 0.785
Grip strength (N/g)	0.083 ± 0.003	0.090 ± 0.002	0.097 ± 0.003	***F*** **_(2, 23)_ = 5.323;** ***P*** **= 0.013**
Activity (a.u)/ 24 h	2504 ± 224	2884 ± 227	4741 ± 317	***F*** **_(2, 23)_ = 21.440;** ***P*** **< 0.001**
Power (% variation)	26.0 ± 0.9	27.0 ± 1.9	35.8 ± 1.1	***F*** **_(2, 23)_ = 17.053;** ***P*** **< 0.001**
Onset (ZT)	12.39 ± 0.19	11.87 ± 0.16	11.93 ± 0.09	*F*_(2, 23)_ = 3.496; *P* = 0.052
Activity in light (%)	25.8 ± 0.9	18.5 ± 1.9	15.8 ± 1.7	***F*** **_(2, 23)_ = 12.451;** ***P*** **< 0.001**

*Maximal forelimb grip strength was measured by a meter during the night and normalize to body weight. Infrared monitors were used to measure total cage activity over a 10-day duration. The average activity levels during a 24-h cycle as shown as a measure of physical activity. The strength of diurnal rhythms in cage activity were assessed using periodogram including power (% variation), onset of activity as well as the % of total activity during the light. Bold values indicate statistically significant genotypic differences. n = 8 per genotype, all males*.

## Discussion

Both clinical and preclinical research indicate that cardiovascular dysfunction should be considered a core symptom of at least in a subset of HD patients (9, 12). Mutant huntingtin (*mHTT*) is ubiquitously expressed so the cardiomyopathy could be the result of deficits in the cardiomyocytes themselves and/or in the dysautonomia driven by the nervous system. In this study, we utilized the BACHD mouse model that allows specific reduction of the *mHTT* expression in the cardiomyocytes when crossed with the *Myh6*-*cre* mouse line (Figure 1). As shown in Figure 2, we achieved a successful reduction in the *mHTT* mRNA and protein levels in the BMYO hearts. The cre-driven recombination would have occurred early in development so that the BMYO mice that we evaluated would have had greatly reduced mHtt from the heart with still high levels of *mHTT* present in the brain. This genetic construct provides a unique model to evaluate the contribution of the *mHTT* in the cardiomyocytes to the CV disease associated with HD.

We examined the CV function and structure using echocardiograms in all three genotypes at 6 months of age. Most of the functional echo parameters did not vary between the genotypes (Table 1). There were two notable exceptions. As previously observed (22), the LV mass of the BACHD hearts was larger than WT (Figure 3A). Hypertrophy is an unusual feature of the BACHD model and contrasts with the reduction in size seen in other models (18, 21, 23, 24). The divergence between the genotypes was already seen at 3 months but was not significant until 6 months of age. This hypertrophy was also observed in the BMYO hearts suggesting that the driver was not the *mHTT* in the cardiomyocytes. In addition, LV ejection fraction (EF) was reduced in the BACHD while the EF of the BMYO heart was comparable to WT levels (Figure 3B). The EF refers to the percentage of blood that is pumped out of a filled ventricle with each heartbeat. It is the relation between the amounts of blood expelled during each cardiac cycle relative to the size of the ventricle. It is important to note that an LV EF of 55% or higher is considered normal under physiologic loading conditions, with an EF of 50% or lower being considered reduced. Thus, the BACHD hearts were still working at functional levels at the 6 month time point. The findings with LV EF were closest to our predictions for a cardiomyopathy driven by *mHTT* in the heart. Perhaps, we needed to wait to an older age to see more robust differences with the echocardiogram. Prior work indicated that the EF of the BACHD hearts steadily declined with age after the 6 months-time point (22) so the present measurements of cardiac output were done at the age of peak performance. Reduced cardiac output are observed in other models including the R6/2 (24) and R6/1 (21) and thus appears to be a general feature of the models (see 8 for review). Recent work described electrocardiogram (ECG) abnormalities in the BACHD line (40) and found several abnormalities associated with increased risk of sudden arrhythmic death in humans (41). Taken together, these data suggest that reduced *mHTT* expression in the cardiomyocytes help improve LV EF in the BMYO mice.

In prior work examining the hearts of middle-aged BACHD mice (>12 months), we found striking fibrosis (22). In the present study, *Col1a* mRNA expression in the BMYO hearts was significantly reduced compared to BACHD (Figure 4A). Similarly, we found that Collagen I immunoreactivity as well as Fibronectin protein expression was highest in the BACHD and reduced in the BMYO heart (Figure 5). In regard to *Mhy7* expression (Figure 4B), the trends are consistent with increased fibrosis as well as an enlarged heart (36, 42, 43) exhibited by the BACHD, and previously described (22). However, the differences were not significant as measured by the one-way ANOVA (Table 2) and the power of the performed test (0.173) is below the desired power of 0.800. Already at this early age, we observed the presence of fibrotic regions in the BACHD and BMYO mouse hearts, but not in the WT (Figure 6). An increase in fibrosis in the heart could explain the reduction in the EF found in the echo data. Histological studies have suggested that myocardial fibrosis can be an important driver of a reduction in EF (44, 45). We also looked at the expression of genes involved in ventricular remodeling including *Gata4* (Figure 4C). These genes are known to be up-regulated in response to injury or cardiomyocyte remodeling (46–48). We found a significant increase in the expression of *Gata4* in the hearts of the BACHD but not in the BMYO line (Table 2). Finally, we examined the expression of *Bnp* (Figure 4D), which is a hormone secreted by cardiomyocytes in the heart ventricles in response to stretching (49, 50). Again, the BACHD mutants exhibited higher expression while the BMYO line showed a much lower expression. Together these data indicate that, at least transcriptionally, the BACHD hearts were under stress that was reduced in the BMYO line.

Finally, we looked at the impact of the cross on motor behaviors that are thought to be reflective of cardiovascular health. In humans, there is strong evidence that grip strength can provide indications of cardiovascular (mal)function (37, 38, 51). For example, a recent study examined the association of objectively measured grip strength (GS) with incident heart failure (HF) (52). Using the UK Biobank dataset, the authors evaluated data from 374,493 individuals. They found that higher GS was associated with 19% lower incidence of HF risk. While these data are correlational, the literature indicates that objective measurements of physical function (GS) are strongly associated with lower HF incidence. In this study, we found that grip strength was reduced in the BACHD compared to WT and that this measure was improved by the excision of the *mHTT* (Figure 7A). Overall, physical activity in this case measured by total activity over 24-hr followed the same pattern (Figure 7B). On the other hand, both of the mutant groups exhibited the reduction in the strength of the rhythm as measured by power (Figure 7C). Most other rhythmic parameters were not improved by the reduction of the *mHTT* from the heart. The central circadian clock is located in the hypothalamus thus improvements in circadian output were not expected from this cross. One unexpected finding is that the inappropriate day-time activity seen in the BACHD line (32, 39) was improved by the cross (Figure 7D). Taken at face value, this result suggests that some of the negative circadian phenotype seen in HD models could be a reflection of compromised CV function. This issue deserves further study.

We were forced to bring this study to a premature closure because of COVID-19. Due to the research stoppage, this work presents several weaknesses. First, while the study was originally designed to longitudinally follow the mice until 12 months of age, we had to terminate the experiments at 6 months. Therefore, we were only able to investigate the role of *mHTT* at early stages of disease progression and, based on our prior work (22), we expected more dramatic effects and differences among genotypes at a later age. In addition, as our colony had to be culled, the sample size for some of the measurements and assays is limited to 3–4 mice per genotype, about half of our original intent. Although we previously showed sex difference in the BACHD mice (53), with the female presenting less severe symptoms at early stages of disease, another limitation of the present study is the usage of only male mice. Future work will need to determine whether the reduction in *mHTT* in the cardiomyocytes differentially affects male and female mice at later stages of the disease, and may ameliorate mitochondrial dysfunction, protein aggregation, ER stress, and/or facilitates autophagy. In the present study, we were only able to interrogate a limited number of pathways. Obviously, because of all the above mentioned limitations, caution should be exerted when interpreting our findings.

In summary, the clinical and preclinical research indicate that cardiovascular dysfunction should be considered a core symptom of at least a subset of HD patients. As established by prior work, there is strong evidence for dysautonomia that can be detected early in the HD progression. In addition, there is evidence for cardiomyopathy and our results suggest that there is cardiac specific pathology in the BACHD pre-clinical model. Reducing *mHTT* specifically from cardiomyocytes did improve the LV EF as measured by the echocardiogram, reduced the expression of several markers of heart disease, and improved a behavioral marker of cardiovascular health. We believe that these findings argue that cardiomyopathy should be considered as part of the consequences of the HD mutation in clinical practice.

## Data Availability Statement

The raw data supporting the conclusions of this article will be made available by the authors, without undue reservation.

## Ethics Statement

The animal study was reviewed and approved by UCLA Animal Research Committee.

## Author Contributions

SP, CG, and CC conceived the hypothesis and experimental design of this study. SP, SL, RB, DW, EC, and MJ performed the experiments. SP, KR, CG, and CC analyzed the data. SP wrote for the first draft. CG and CC edited, wrote, and compiled the final version paper with contribution of the other authors. All authors contributed to the article and approved the submitted version.

## Funding

Core equipment used in this study was supported by the National Institute of Child Health Development under Award Number: 5U54HD087101.

## Conflict of Interest

The authors declare that the research was conducted in the absence of any commercial or financial relationships that could be construed as a potential conflict of interest.

## Publisher's Note

All claims expressed in this article are solely those of the authors and do not necessarily represent those of their affiliated organizations, or those of the publisher, the editors and the reviewers. Any product that may be evaluated in this article, or claim that may be made by its manufacturer, is not guaranteed or endorsed by the publisher.
